# The impact of maxillary dimensions on determining surgical approach of fungal ball in the maxillary sinus

**DOI:** 10.1038/s41598-024-58726-z

**Published:** 2024-05-04

**Authors:** Young-Ha Lee, Won Ki Cho, Dong Hyun Kim, Ji Heui Kim

**Affiliations:** 1 Department of Otorhinolaryngology – Head and Neck Surgery, Asan Medical Center, University of Ulsan College of Medicine, 88 Olympic-Ro 43-Gil, Songpa-Gu, Seoul, 05505 Republic of Korea; 2 University of Ulsan College of Medicine, Seoul, Republic of Korea

**Keywords:** Bone, Fungal infection, Computed tomography, Therapeutic endoscopy

## Abstract

Endoscopic middle meatal antrostomy (MMA) is commonly used for maxillary sinus (MS) fungal ball removal. For challenging cases involving anterior or inferior recess, an additional inferior meatal approach (IMA) might be needed. We analyzed the differences in MS dimensions on CT scans according to the surgical approach to suggest preoperative variables that could facilitate an additional IMA. CT scans of 281 adult patients who underwent ESS for the MS fungal ball (139 MMA, 62 MMA & IMA) were evaluated for comparative analysis of 8 MS measurements based on the surgical approach. Complete removal was achieved in all cases. Age and sex didn't differ significantly (*p* > 0.05). The maximum distances between the anterior–posterior walls, the inferior ostium border to the lateral recess, and the ostium to the inferior wall of the MS were statistically greater in the MMA & IMA group compared to the MMA group (*p* = 0.003, *p* = 0.005, and *p* = 0.010, respectively), especially among females. This study underscores the clinical importance of specific measurements—anterior to posterior wall, medial wall to lateral recess, and ostium to inferior wall of the maxillary sinus—for guiding optimal surgical approaches in MS lesions.

## Introduction

The incidence of fungal sinusitis has increased over several decades owing to the increased diagnostic rates of imaging modalities and a population with underlying chronic disease^1^. Endoscopic sinus surgery (ESS) is the gold standard of treatment for fungal ball. Deposits and colonization of inhaled fungus impair mucociliary function, obstruct the ostiomeatal complex, and decrease ventilation. Therefore, the goals of surgical treatment are the complete removal of fungal debris and the re-establishment of proper ventilation and drainage.

Approximately 90% of fungal balls are found in the maxillary sinus (MS)^2,3^. Middle meatal antrostomy (MMA) is the primary method for exposing MS; however, the accessibility to reach the surface area and volume of MS through MMA is limited. Therefore, additional approaches, such as inferior meatal antrostomy (IMA), canine fossa, extended middle meatal antrostomy, endoscopic maxillary mega-antrostomy, modified endoscopic Denker’s approach, endoscopic medial maxillectomy, and endoscopic modified medial maxillectomy (termed the pre-lacrimal approach) have been used to visualize the anterior and inferior maxillary recess, usually invisible through conventional MMA^4,5^. In particular, IMA can be easily performed without external incision and complications, and has been suggested as one of the best approaches to access the anterior wall of the MS^6^.

Given that the purpose, methods, and complications of an additional extended approach besides conventional MMA should be explained to the patient before surgery, specific parameters that can predict the need for them in advance are warranted. Therefore, this study aimed to analyze the dimensions of MS on computed tomography (CT) scans, which required IMA in addition to MMA, by comparing their difference between an MMA-only group and MMA and IMA group, and to suggest specific parameters on CT scans that can predict the need for an additional approach in the patients with MS fungal ball.

## Results

The dimensions of male patients were larger than those of female patients. The maxillary sinus height (MSH), ostium height (OsH), maximum oblique width (Max OW), maximum horizontal width (Max HW), and distance from ostium to maxillary sinus inferior wall (OsI) were found to be significantly greater in males than females (*p* < 0.001, *p* = 0.008, *p* = 0.001, *p* = 0.001, and *p* = 0.002, respectively, Table 1). The maximum anteroposterior distance of the MS (Max AP), Max OW, and OsI were significantly greater in the MMA and IMA groups than in the MMA group (*p* = 0.003, *p* = 0.005, and *p* = 0.010, respectively, Table 2).

**Table 1:** Comparisons of means for parameters of maxillary sinus dimensions according to sex.

Maxillary sinus dimensions Mean ± SD (mm)	Male (*n* = 75)	Female (*n* = 126)	*p*-value^a^
MSH	36.03 ± 5.39	33.13 ± 5.70	< 0.001*
OsH	25.51 ± 5.24	23.30 ± 5.96	0.008*
NFH	6.92 ± 3.94	6.86 ± 4.58	0.916
Max AP	34.87 ± 4.11	33.88 ± 4.24	0.108
Max ARD	12.93 ± 3.40	13.00 ± 3.32	0.883
Max OW	30.14 ± 5.02	27.78 ± 4.56	0.001*
Max HW	25.80 ± 4.78	23.53 ± 4.59	0.001*
OsI	28.81 ± 4.56	26.61 ± 5.03	0.002*

*MSH* maxillary sinus height, *OsH* ostium height, *NFH* nasal floor height, *Max AP* maximum anteroposterior distance, *Max ARD* maximum anterior recess depth, *Max OW* maximum oblique width, *Max HW* maximum horizontal width, *OsI* distance from ostium to maxillary sinus inferior wall.

^a^Independent-sample *t*-test was performed, *p-value < 0.05.

**Table 2:** Comparisons of means for parameters of maxillary sinus dimensions according to endoscopic surgical approaches.

Maxillary sinus dimensions (Mean ± SD, mm)	MMA (*n* = 139)	MMA & IMA (*n* = 62)	*p*-value^a^
MSH	34.00 ± 5.66	34.70 ± 5.97	0.425
OH	23.78 ± 5.57	24.89 ± 6.22	0.212
NFH	6.53 ± 4.21	7.67 ± 4.56	0.084
Max AP	33.66 ± 4.35	35.56 ± 3.57	0.003*
Max ARD	13.10 ± 3.47	12.69 ± 3.04	0.422
Max OW	28.03 ± 4.85	30.08 ± 4.61	0.005*
Max HW	24.23 ± 4.94	24.70 ± 4.42	0.524
OsI	26.83 ± 5.03	28.78 ± 4.56	0.010*

*MSH* maxillary sinus height, *OsH* ostium height, *NFH* nasal floor height, *Max AP* maximum anteroposterior distance, *Max ARD* maximum anterior recess depth, *Max OW* maximum oblique width, *Max HW* maximum horizontal width, *OsI* distance from ostium to maxillary sinus inferior wall.

^a^Independent-sample *t*-test was performed, *p-value < 0.05.

## Discussion

This study evaluated the preoperative parameters on CT scans that could predict the need for an additional IMA approach to MMA for the endoscopic removal of MS fungal ball. Patients who had their MS fungal ball removed via IMA in addition to MMA had greater distances from the ostium to the lower wall, from the ostium to the lateral recess, and from the anterior wall to the posterior wall of the maxillary sinus than patients who had their MS fungal ball removed via MMA alone. These results suggest that in patients with large distances in the MS, adding IMA to MMA is likely required to completely remove the MS fungal ball.

Paranasal sinus fungal ball is a non-invasive chronic fungal sinusitis without allergic mucin, containing a clump of mold in the paranasal sinuses^7^. En-bloc removal of the fungal ball is almost impossible. They are entangled in a brittle, cheese-like substance and often accompanied by severe inflammatory mucosal edema and purulent discharge. The principle of fungal ball surgery is to ensure adequate visibility during ESS to completely remove these substances and to completely flush out the affected sinuses so that no fungal debris remains to prevent recurrence. MS fungal balls, which correspond to the location of most fungal balls^2,3,7,8^, are primarily removed with ESS and MMA. However, several studies have suggested that endoscopic MMA alone has limitations in observing the entire MS^9–11^. This visual limitation of MMA seems to be due to the anatomical features of the MS. General MMA is performed at the posteromedial portion of the MS by removing the membranous portion between the anterior and posterior fontanelle after uncinectomy^12^. In addition, the angle of access to the anterior inferior wall, the anterior medial wall, and the prelacrimal recess of the MS through MMA requires a visual direction change of more than 120 degrees based on the direction of the initial access path^11^. Another study confirmed that it is difficult to identify the pre-lacrimal recesses of the MS with endoscopic MMA alone^13^. Even with 70° endoscopy, the MS may not be sufficiently visualized with MMA alone. The recurrence rate of MS fungal ball removed with MMA alone is between 3 and 14%, indicating that such inadequate visualization may leave fungal debris and increase the risk of MS fungal ball recurrence with incomplete surgery^7,9,14^.

Various additional approaches have been utilized to overcome the limitations of the visualization of endoscopic MMA. External approaches (Caldwell-Luc surgery and canine fossa approaches) may be inadequate for the non-invasive treatment of fungal balls as they can lead to various complications, such as trigeminal nerve palsy, facial pain, and fistula^5,15^. Extended endoscopic approaches to widen the MMA to obtain improved visualization, such as extended MMA, endoscopic maxillary mega-antrostomy, modified endoscopic Denker's approach, and endoscopic medial maxillectomy, have also been proposed^7,9,16,17^. However, these are very harsh approaches for the treatment of non-invasive fungal balls, and are difficult to advocate for as the first-choice alternative approach due to the excessive damage to surrounding normal tissues and the lengthening of the operation time. A gauze-assisted technique was proposed to overcome the short visualization; however, it has a fundamental limitation in that remaining fungal debris cannot be completely identified^18^. The pre-lacrimal approach is an excellent surgical method with low aggressiveness, but it could not always be viable due to the anatomical variation of the lacrimal duct and pre-lacrimal recess. It also has the drawback of having a risk of potential lacrimal duct damage^19^.

IMA, comprising part of the classic Caldwell-Luc surgery, was also proposed in this regard^14^. IMA offers reasonable additional visualization despite a straightforward, quick, and low-bleeding procedure. IMA seems to be an effective option for visualizing the anterior inferior wall and medial inferior wall of MS without unnecessary damage. Although IMA is controversial due to its impaired MS mucociliary clearance, risk of lacrimal duct injury, and high spontaneous occlusion rate, it nonetheless seems the most preferred option for further visualization after MMA^9^. Despite concerns, IMA is known to rarely damage the nasolacrimal duct, as it approaches through areas where the bone is thicker and the meatus height is lowered near Hasner's valve, thus reducing the possibility of injury^18^. Some even maintain that MMA is not mandatory for all MS conditions, suggesting that IMA alone can effectively address MS diseases^14^. Considering the findings from a recently published meta-analysis on the treatment of paranasal sinus fungal balls, which identified IMA as the most commonly employed alternative following MMA, MMA still appears to remain the primary surgical approach to address MS fungal balls^20^. A combination of MMA and IMA is reportedly required in more than 60% of ESS procedures for fungal sinusitis^11^.

Various attempts to improve accessibility to the MS have led to studies predicting the accessibility of MMA. A previous study using four cadavers quantitatively demonstrated that a single or combination of microdebrider curvature provided access of approximately 80% of the volume and mucosal surface area to the entire MS mucosa via MMA through image standardization of MS parameters^6^. However, studies have quantitatively confirmed accessibility through MMA by measuring parameters on CT scans, directly evaluating the anatomical MS parameters that require extended approaches other than MMA. Paranasal sinus CT is a general preoperative examination for fungal ball surgery. Understanding the MS parameters that facilitate IMA addition has the advantage of predicting the possibility of performing IMA during surgery, allowing a more accurate surgical method to be explained to the patient before the surgery. Furthermore, it is possible to predict the operation time and prepare the necessary tools for surgery. Finally, the planned approach also has the advantage of preventing postoperative complications related to the nasolacrimal duct and inferior meatus. Therefore, this study analyzed the MS parameters on CT scans that require IMA, often combined with MMA, to predict in advance the cases in which IMA is needed. Previously reported sex differences in the parameters of the MS were also observed within this study^21^. As the Max OW, the Max AP, and the OsI increased, there was a tendency to combine IMA. This is probably because it is difficult to completely remove the fungal ball with MMA alone in cases where the anatomical structure of the anterior recess depth is relatively deep compared to the size of the MS or in the case of MS where the distance from the ostium to the deepest part is long. Furthermore, these findings suggest that preoperative CT assessments of the maximum oblique width of the MS, maximum AP distance of the MS, and the distance from the ostium to the MS inferior wall can serve as indicators to predict the likelihood of needing to complement MMA with IMA.

This is a retrospective, single-center study, which is by the variables depending on the preference and technique of the operator and the anatomical variation of the patient when selecting an IMA. However, our study holds the advantage in that there was no intentional bias intervention of the operator in selecting IMA. A more unified review of the variables affecting accessibility should be devised depending on the nuances in the methods, size, and location of IMA. In addition to the MS parameters used in this study, designing anatomical dimensions that can be easily measured and used clinically is required. Incorporating preoperative CT scan insights to not only determine IMA's necessity but also to assess procedural challenges could pave the way for novel methodologies that predict IMA's complexities. Notably, incorporating insights from preoperative CT scans—specifically regarding the thickness of the medial wall—enables the preemptive preparation for the use of a drill if necessary. Such advancements in predictive analytics could refine the decision-making for IMA, leading to more nuanced surgical planning in ESS for MS. Since the study was conducted as a result of the treatment of patients without recurrence of fungal balls, applications of these results to sinusitis or tumor require additional consideration.

This study showed that distances from the anterior to the posterior wall of the MS and the ostium to the inferior and lateral walls are clinically significant determinants in choosing IMA as an additional approach after MMA for MS fungal ball treatment. These results may be helpful in consulting patients with MS fungal balls and in preparing and planning surgery.

## Methods

### Study patients and surgical techniques

This study included 201 patients (75 males and 126 females; mean age: 60.47 years; range: 25–82 years) who had fungal balls in the unilateral MS, underwent paranasal sinus CT scans, and were successfully treated with ESS between March 2013 and December 2018. The presence of fungal balls was confirmed histopathologically. Patients who were younger than 20 years old, who had fungal balls in the ethmoid, sphenoid, or frontal sinuses, or were diagnosed with allergic fungal sinusitis or invasive fungal sinusitis were excluded. This study was approved by the institutional review board of Asan Medical Center, exempting the study from requiring patient informed consent (2020–1673).

All patients underwent MMA or MMA combined with IMA (MMA and IMA) without an external approach. Fungal ball in the MS was removed through MMA alone in 139 patients (69.2%) and MMA and IMA in 62 patients (30.8%). The selection principle of IMA hinges on the accessibility and thoroughness of fungal ball removal. If fungal debris is visible during 70° endoscopic evaluation but instrumentation through the MMA is ineffective in reaching the debris or complete removal of the fungal debris cannot be confirmed, IMA intervention is deemed necessary.

The surgical procedures were executed by two otorhinolaryngology specialists, J.H.K. and B.J.L, at our institution. Their surgical techniques ensured a consistent and unified surgical approach. The MMA technique involved dilating the maxillary sinus fontanelle sufficiently to enable maximum visualization of the MS interior using a 70° endoscope. IMA is positioned posteriorly, inferiorly, and horizontally beneath Hasner's valve. Starting with the precise localization of Hasner’s valve using a 30° endoscope, a crucial step for reducing the risk of injury to the nasolacrimal duct, a curved 4 mm trocar is then employed to penetrate the thinnest area of bone at the superior aspect of the inferior meatus, creating an entry point into the antrum. In cases where the preoperative CT indicated a thickened medial wall, a drill was prepared in advance and used when needed. Subsequently, a window approximately 2 cm long and 1 cm high is carefully fashioned with side-biting forceps to provide sufficient access to the MS. Notably, none of the patient who underwent IMA had injuries to the Hasner’s valve or nasolacrimal duct. Both MMA and IMA were performed at separate anatomical locations and no inferior turbinate injuries were observed.

### Dimensions

**Figure 1:**
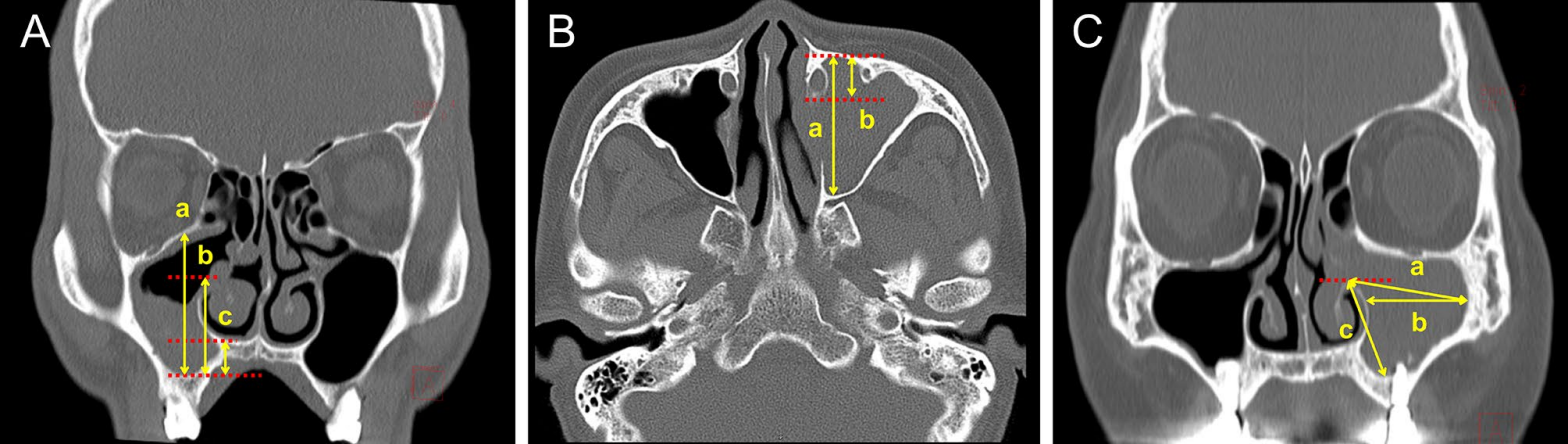
Parameters of maxillary dimensions. (**A**) Several parameters of maxillary dimensions were measured in PNS CT or OMU CT, estimated as (**a**) Maxillary sinus height (MSH), (**b**) Ostium height (OsH), and (**c**) Nasal floor height (NFH) in the coronal view. (**B**) (**a**) MS maximum anteroposterior distance (Max AP) and (**b**) Maximum anterior recess depth (Max ARD) were calculated in the axial view. (**C**) Estimation of (**a**) Maximum oblique width (Max OW), (**b**) Maximum horizontal width (Max HW), and (**c**) Distance from ostium to maxillary sinus inferior wall (OsI) was depicted in coronal view.

Eight dimensions in coronal and axial images of preoperative CT scans were measured (Fig. 1). MS height (MSH) defines the vertical distance from the most inferior wall to the superior wall of MS in coronal view (Fig. 1A–a); ostium height (OsH) defines the distance from the inferior wall of MS to the inferior border of the ostium level (Fig. 1A–b); nasal floor height (NFH) defines the distance from the inferior wall of MS to the nasal floor (NF) (Fig. 1A–c); maximum AP distance (Max AP) defines the longest length from the anterior to the posterior distance of MS in axial view (Fig. 1B–a); maximum anterior recess depth (Max ARD) defines the longest length from the anterior recess to the nasolacrimal duct (NLD) in axial view (Fig. 1B–b); maximum oblique width (Max OW) defines the longest oblique distance from the inferior border of the ostium to the lateral recess in the coronal view (Fig. 1C–a); maximum horizontal width (Max HW) defines the longest distance from lateral recess to the medial wall of the maxillary sinus (MS) in coronal view (Fig. 1C–b); and distance from the ostium to the maxillary sinus inferior wall (OsI) defines the oblique distance from the inferior border of the ostium to the inferior wall of MS (Fig. 1C–c).

### Statistical analyses

Data are expressed as mean ± standard deviations for quantitative variables. Comparisons between groups were performed using an independent sample *t*-test for quantitative variables. SPSS version 20 software (Armonk, NY, USA) was used for statistical analysis, and a* P*-value of less than 0.05 was considered to indicate statistical significance.

### Ethical approval

This study was conducted in accordance with the Declaration of Helsinki. This study was approved by the institutional review board of Asan Medical Center, exempting the study from requiring patient informed consent (2020–1673).

## Data Availability

The datasets generated during and/or analysed during the current study are available from the corresponding author on reasonable request.
